# Supplementary material for: Potential of mean force between like-charged nanoparticles: Many-body effect
Source: Sci Rep. 2016 Mar 21;6:23434. doi: 10.1038/srep23434 (PMC4800448; doi:10.1038/srep23434)
Supplement: Supplementary Information [file srep23434-s1.pdf]

Supplementary Information for

Potential of mean force between like-charged nanoparticles: Many-body effect

Xi Zhang[1†], Jin-Si Zhang[1†], Ya-Zhou Shi[1], Xiao-Long Zhu[2], and Zhi-Jie Tan[1*]

[1] *Center for Theoretical Physics and Key Laboratory of Artificial Micro & Nano-structures of Ministry of Education, School of Physics and Technology, Wuhan University, Wuhan 430072, China*

[2] *Department of Physics, School of Physics & Information Engineering, Jianghan University, Wuhan 430056, China*

---

† These authors contributed equally to the work

* To whom correspondence should be addressed: zjtan@whu.edu.cn

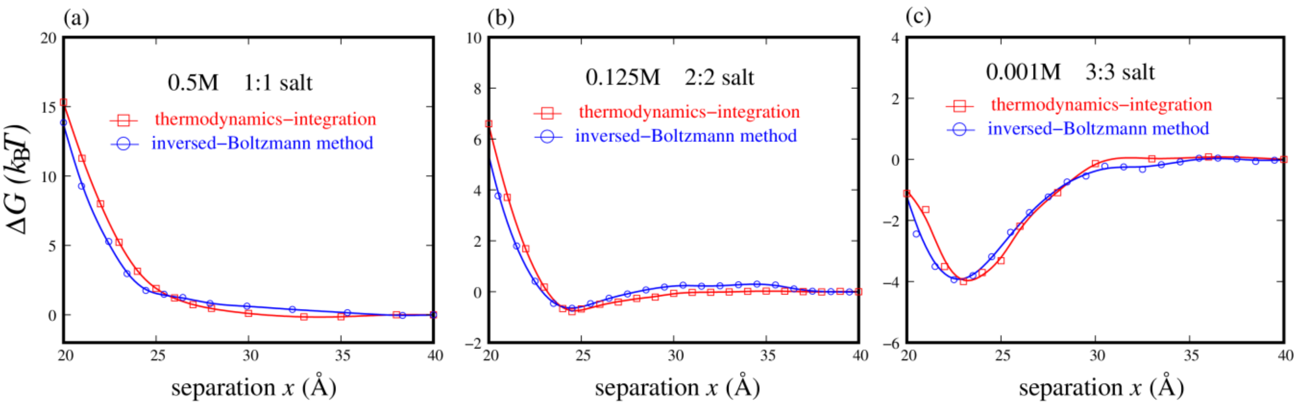

Figure S1. The comparisons between thermodynamics-integration (TI) method (red square) and inversed-Boltzmann method (blue circle) [Y. Y. Wu *et al.*, Phys. Lett. A **377**, 1911-1919 (2013)]. The potentials of mean force ΔG_x as functions of the separation x between nanoparticles with -20e for the two-body systems in 0.5M 1:1 salt solution (a), 0.125M 2:2 salt solution (b) and 0.001M 3:3 salt solution (c).

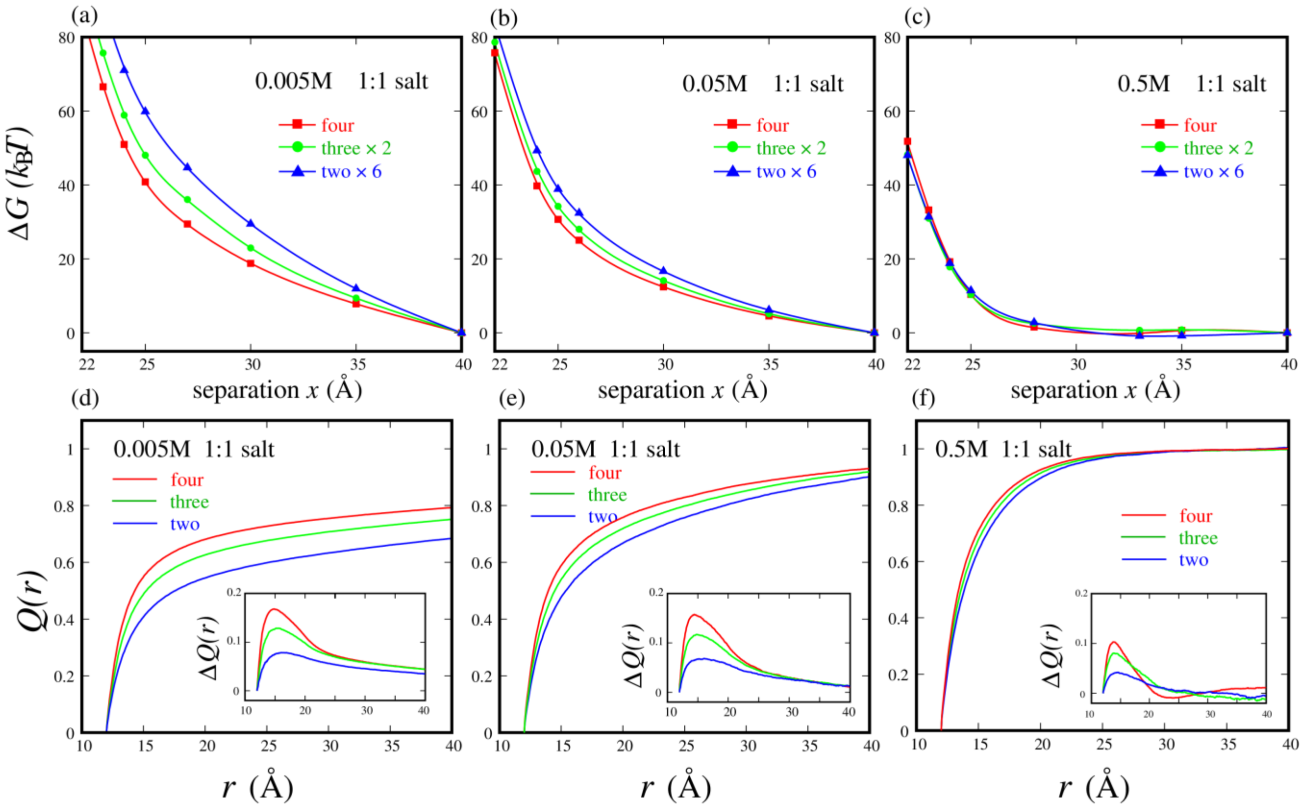

Figure S2. (a-c) The potentials of mean force ΔG_x as functions of the separation x between nanoparticles with -20e for the four-body systems in 1:1 salt solutions which are calculated respectively by the pair-wise potential of mean force abstracted from two-body, three-body and four-body systems (denoted respectively by two nanoparticles×6, three nanoparticles×2, and four nanoparticles). (a) 0.005M, (b) 0.05M, and (c) 0.5M. (d-f) Net charge distribution $Q(r)$ per unit charge on nanoparticles as a function of distance r around the nanoparticles with x=25Å in 0.005M (d), 0.05M (e) and 0.5M (f) 1:1 salt solutions. The insets show the increase of $Q(r)$ due to the approaching of nanoparticles from x=40Å to x=25Å for the systems of two nanoparticles, three nanoparticles and four nanoparticles.

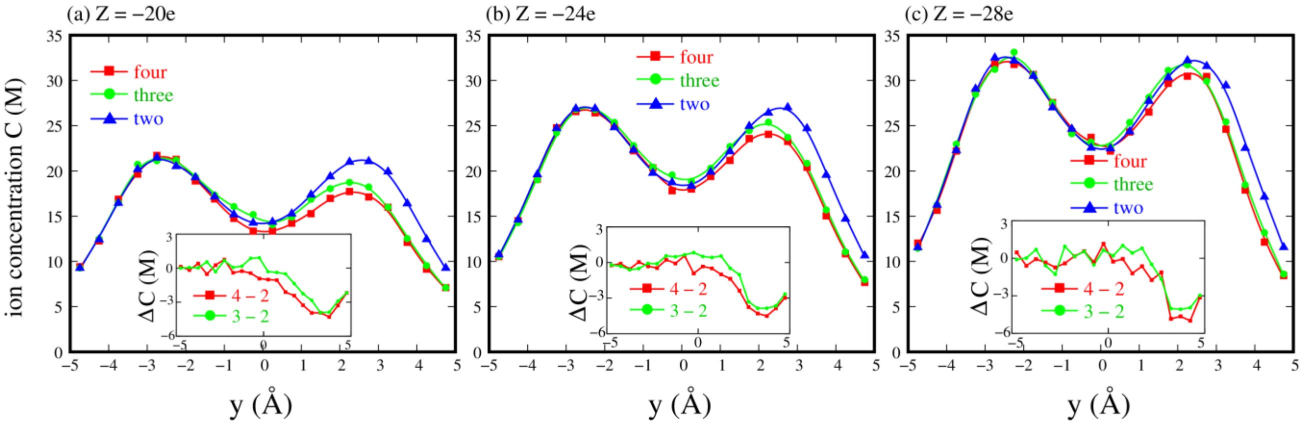

Figure S3. (a-c) The detailed ion concentration distribution of "ion bridge" between nanoparticles with x=24Å in 0.01M 2:2 salt solutions for two-body, three-body and four-body nanoparticles with Z=-20e (a), Z=-24e (b) and Z=-28e (c). The insets show the concentration differences between four-body and two-body systems (red; denoted by 4–2) as well as those between three-body and two-body systems (green; denoted by 3–2). The similar curves for x=25Å are shown in Fig. 5.

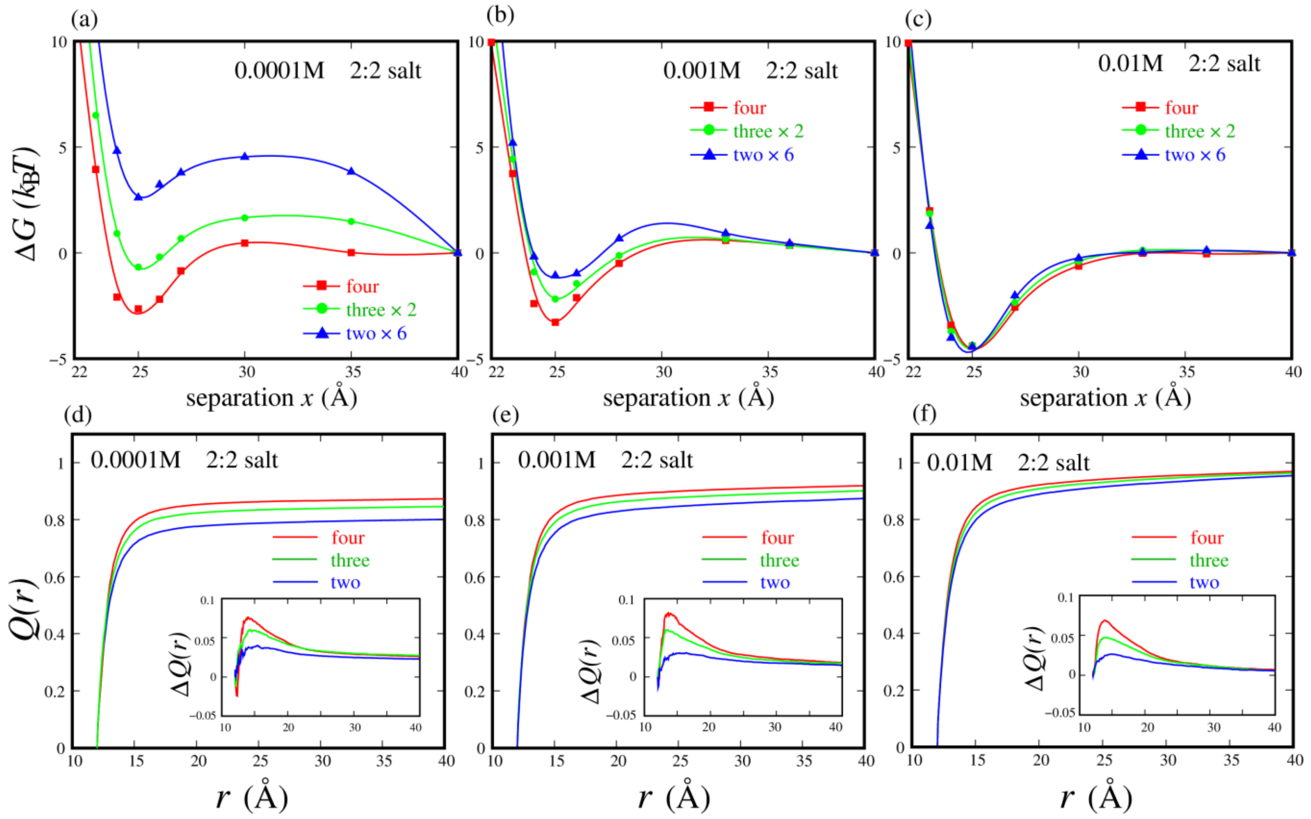

Figure S4. (a-c) The potentials of mean force ΔG_x as functions of the separation x between nanoparticles with -20e for the four-body systems in 2:2 salt solutions which are calculated respectively by the pair-wise potential of mean force abstracted from two-body, three-body and four-body systems (denoted respectively by two nanoparticles×6, three nanoparticles×2, and four nanoparticles). (a) 0.0001M, (b) 0.001M, and (c) 0.01M. (d-f) Net charge distribution $Q(r)$ per unit charge on nanoparticles as a function of distance r around the nanoparticles with x=25Å in 0.0001M (d), 0.001M (e) and 0.01M (f) 2:2 salt solutions. The insets show the increase of $Q(r)$ due to the approaching of nanoparticles from x=40Å to x=25Å for the systems of two nanoparticles, three nanoparticles and four nanoparticles.
